# Biochar Beyond Soil Amendment: Emerging Biological, Environmental, and Biomedical Properties and Applications

**DOI:** 10.3390/molecules31203648

**Published:** 2026-10-13

**Authors:** Sidra Amin, Agnieszka Śmieszek, Sebastian Opaliński, Magdalena Wołoszyńska

**Affiliations:** 1 Department of Genetics, Faculty of Biology and Animal Sciences, Wroclaw University of Environmental and Life Sciences, ul. Kozuchowska 7, 51-631 Wroclaw, Poland; 2 Laboratory of Preclinical Research “In VetBio”, Applied Biology Center, Department of Pharmacology and Toxicology, Faculty of Veterinary Medicine, Wroclaw University of Environmental and Life Sciences, Norwida 31, 50-375 Wroclaw, Poland; 3 Department of Environment, Animal Hygiene and Welfare, Wroclaw University of Environmental and Life Sciences, Chelmonskiego 38C, 51-630 Wroclaw, Poland; 4 Department of Cellular Molecular Biology, Faculty of Biotechnology, University of Wroclaw, Joliot-Curie 14a, 50-383 Wroclaw, Poland

**Keywords:** biochar, adsorption, electron transfer, microbial habitat, biomolecule binding, mineral precipitation, biological responses

## Abstract

Biochar is increasingly being explored beyond its established environmental and agricultural uses as a functional material that interacts with microorganisms, cells, and biological molecules. However, biochar is not a uniform material, and its biological effects cannot be interpreted independently of feedstock composition, production conditions, particle size, surface chemistry, and post-production modification. This review synthesizes current knowledge on the relationships between these physicochemical characteristics and the biological responses associated with biochar and biochar-based materials. This review examines how specific physicochemical characteristics of biochar relate to interaction mechanisms and biological responses, while identifying areas where mechanistic evidence remains limited. Particular attention is given to adsorption, electron transfer, microbial habitat formation, biomolecule binding, and mineral precipitation as mechanisms that may underlie changes in oxidative balance, antimicrobial activity, animal physiology, cellular responses, and tissue regeneration. The review also critically considers emerging nanobiochar and engineered composites investigated as drug carriers, wound-healing materials, regenerative scaffolds, and platforms for anticancer therapy. Importantly, reported effects often depend on highly heterogeneous materials and experimental models, and the biological contribution of the biochar matrix is not always clearly distinguished from that of incorporated metals, polymers, drugs, or other functional components. Current evidence therefore supports biochar as a tunable platform rather than a universally bioactive material. Its successful development for biological and biomedical applications will require standardized production and characterization as well as mechanism-oriented experimental designs.

## 1. Introduction

Biochar is a carbon-rich material produced when biomass is heated in a closed container under limited-oxygen or no-oxygen conditions. It can be produced from a wide variety of feedstocks, such as wood, manure, or crop residues [1]. Biochar has a long history, as Amazonian Indians used it to produce the terra preta soils of the Amazon Basin, which, 1000 years after their creation, remain more fertile than surrounding lands [2]. Biochar has diverse potential applications, including carbon sequestration, soil fertility improvement, pollution remediation, and the valorization and recycling of agricultural residues [3,4,5]. In recent years, interest in biochar and its research has increased significantly [6] because it plays an important role in addressing several pressing environmental issues, including climate change [7], soil degradation [8,9], and waste management [10]. However, global biochar production statistics are highly uncertain. Available data mainly rely on incomplete industry surveys and coarse-aggregated datasets rather than standardized, comprehensive reporting frameworks [11,12].

Rather than focusing on individual applications, this review examines how biochar physicochemical properties relate to key interaction mechanisms, including adsorption, electron transfer, microbial habitat formation, and biomolecule interactions, and how these mechanisms contribute to biological responses. This structure–property–function perspective also provides a basis for critically evaluating emerging nanobiochar and engineered biochar-based materials and the extent to which their reported effects can be attributed to the biochar matrix.

Biochar is most commonly produced through pyrolysis at different temperatures, typically ranging from 300 to 700 °C. Aside from temperature, another key variable in biochar production is residence time, which typically ranges from minutes to several hours. These two factors play an important role in determining fixed-carbon content, pH value, the amount of acidic and basic functional groups, biochar yield, and adsorption capacity [13]. Different thermochemical conversion pathways may also be involved in biochar production, including slow pyrolysis [14], fast pyrolysis [15], microwave pyrolysis [16], vacuum pyrolysis [17], and gasification (co-gasification) [18]. The final properties of biochar depend upon physicochemical properties, including feedstock type, temperature, heating rate, and residence time, which collectively affect surface area, porosity, ash content, functional groups, and mineral composition [1]. These properties influence biochar suitability for environmental applications and its interactions with biological systems, including microbial colonization [19], biomolecule adsorption [20], and redox-mediated processes [21].

Although these relationships have often been described qualitatively, several original experimental studies have demonstrated quantitative associations between specific biochar characteristics and biological responses. Biochar particle size and application rate were shown to affect sub-lethal responses in earthworms, with smaller particles (<0.5 mm) and higher application rates (6%) posing sub-lethal toxicity under the tested conditions [22]. Similarly, biochar cytotoxicity was found to be associated with concentration- and time-dependent effects in mammalian fibroblast cells (NIH 3T3 mouse fibroblast cell line), where EC_10_ values were 49.6 and 18.8 μg/mL after incubation for 24 and 48 h, respectively. [23]. Changes in the soil pore structure (increase in the total porosity and pore volumes of soil) induced by the application of biochar have also been linked quantitatively (soil pores of >5 µm) with positive effects on the microbial diversity and abundance (of aerobic bacterial genera and fungal genera) [24]. The differences among particle-size fractions have been shown to alter contaminant (Cadmium) speciation, transformation, and associated ecotoxicity, where colloidal-BC exhibited the highest ecotoxicity (in comparison to macro- or nanobiochar) for *Scenedesmus obliquus*. [25]. Collectively, these findings indicate that biochar physicochemical characteristics can influence biological responses through measurable, but context-dependent, relationships. However, because feedstock, pyrolysis conditions, particle characteristics, surface chemistry, and application rate frequently vary simultaneously among biochars, the current evidence is better regarded as comparative and system-specific rather than as a generalized quantitative structure–activity relationship. Overall, quantitative evidence exists for individual physicochemical property–biological response relationships, but a generalized quantitative structure–activity relationship has not yet been established for biochar.

Biochar has a vast range of applications in agriculture, environmental engineering, and livestock systems [2]. In agricultural systems, biochar addition can improve soil physical properties, including aggregate stability and water retention, while also increasing nutrient availability, microbial activity, and under appropriate conditions, crop productivity [26,27,28]. Long-term field experiments further demonstrate that biochar application can increase soil organic carbon stocks, supporting its potential for long-term carbon sequestration [29].

Biochar possesses high chemical stability and sorptive properties that contribute to its applications in nutrient retention and greenhouse-gas mitigation [30], waste management, and pollutant removal [31]. Its persistent carbon structure also enables long-term carbon sequestration [32]. In environmental engineering, biochar has attracted considerable attention as a low-cost adsorbent for contaminant remediation (volatile organic compounds (VOCs)) [31]. Experimental studies have demonstrated that biochar can sorb organic contaminants and immobilize inorganic contaminants such as heavy metals (Cd and Pb) in soil, supporting its application in soil remediation strategies [31,33,34].

Recent work further highlights the influence of pyrolysis conditions on carbon materials used for environmental purification. Ren et al. [35] showed that lignosulfonate-derived porous carbon prepared at 800 °C exhibited increased surface area, graphitization, and electrical conductivity, and achieved 95.3% tetracycline degradation when coupled with peroxydisulfate activation, illustrating how production conditions can influence pollutant-removal performance.

The evidence supporting adsorption, electron transfer, microbial habitat formation, and biomolecule binding mechanisms differs according to the biological context. Biochar-mediated adsorption and immobilization of organic and inorganic contaminants have been directly demonstrated in environmental systems [31,33,34], whereas direct biological evidence is available for the microbial colonization of biochar surfaces and pores and for relationships between biochar pore characteristics and microbial community responses [24,36]. Biochar-mediated extracellular and interspecies electron transfer has also been demonstrated experimentally in microbial systems [37,38]. In addition, the adsorption of biological macromolecules, including extracellular enzymes, has been experimentally observed [39] and linked to biochar surface and pore characteristics [40]. Thus, although these mechanisms have each been investigated experimentally, the strength and biological relevance of the evidence vary among mechanisms and experimental systems.

In livestock and veterinary applications, the use of biochar as a feed additive enhances animal growth, hematological profiles, meat, milk, and egg yields, resistance to illnesses (especially gut-pathogenic bacteria), and reduces ruminant methane emissions. Due to the strong sorption capacity of biochar, it can also efficiently remove contaminants and poisons from the animals’ bodies and the surroundings of the farm in which animals are raised [41].

More recently, advances in nanobiochar engineering have expanded the potential applications of biochar into biomedical fields, including targeted drug delivery and cancer therapy [42]. The wide range of agronomic and environmental benefits associated with biochar has contributed to its recognition not only as a soil amendment, but also as a potential carbon dioxide removal strategy through the long-term sequestration of biomass-derived carbon in soils [43]. Biochar is a chemically stable carbonaceous material with an aromatic structure [44] that resists microbial decomposition over extended periods, enabling a substantial fraction of its carbon to persist in soils for centuries under appropriate conditions [45,46]. In this way, biochar can function as a long-term terrestrial carbon sink by promoting the persistence and sequestration of carbon in soils, thereby contributing to climate change mitigation [47]. On the other hand, recent studies indicate that biochar is not universally beneficial and requires careful management and quality control. Despite biochar’s potential benefits, concerns remain about its long-term environmental safety and effects on soil organisms because biochar properties and their interactions with soil systems can change over time, and some ecological effects and underlying mechanisms remain insufficiently understood [48,49]. An in-depth understanding of the environmental fate of biochar-derived dissolved and particulate carbon in freshwater and terrestrial systems is needed, as biochar-derived compounds and particles may pose ecological risks and potentially diminish some of the anticipated climate benefits of biochar application [50,51]. Biochar properties vary substantially depending on feedstock type and pyrolysis temperature, particle size, surface chemistry, and post-treatment, and these differences can influence soil organic matter mineralization and microbial community structures [52]. Therefore, inadequate characterization of biochar properties may complicate the prediction of its effects on soil microbial communities and functions. To facilitate a comparison among studies, the characteristics of the biochars used in the studies discussed in this review, including feedstock, pyrolysis temperature, modification, and dose or application rate, are summarized where reported in a table as File S2. Parameters that were not reported in the original studies are indicated as “NR: not reported”.

Biochar addition can increase the sorption of soil-applied herbicides and reduce their bioavailability and efficacy, potentially compromising weed control and increasing the need for additional herbicide or weed-management interventions [53]. Accordingly, reported effects are considered in relation to biochar characteristics and experimental conditions rather than being generalized to biochar as a uniform material.

**Figure 1:**
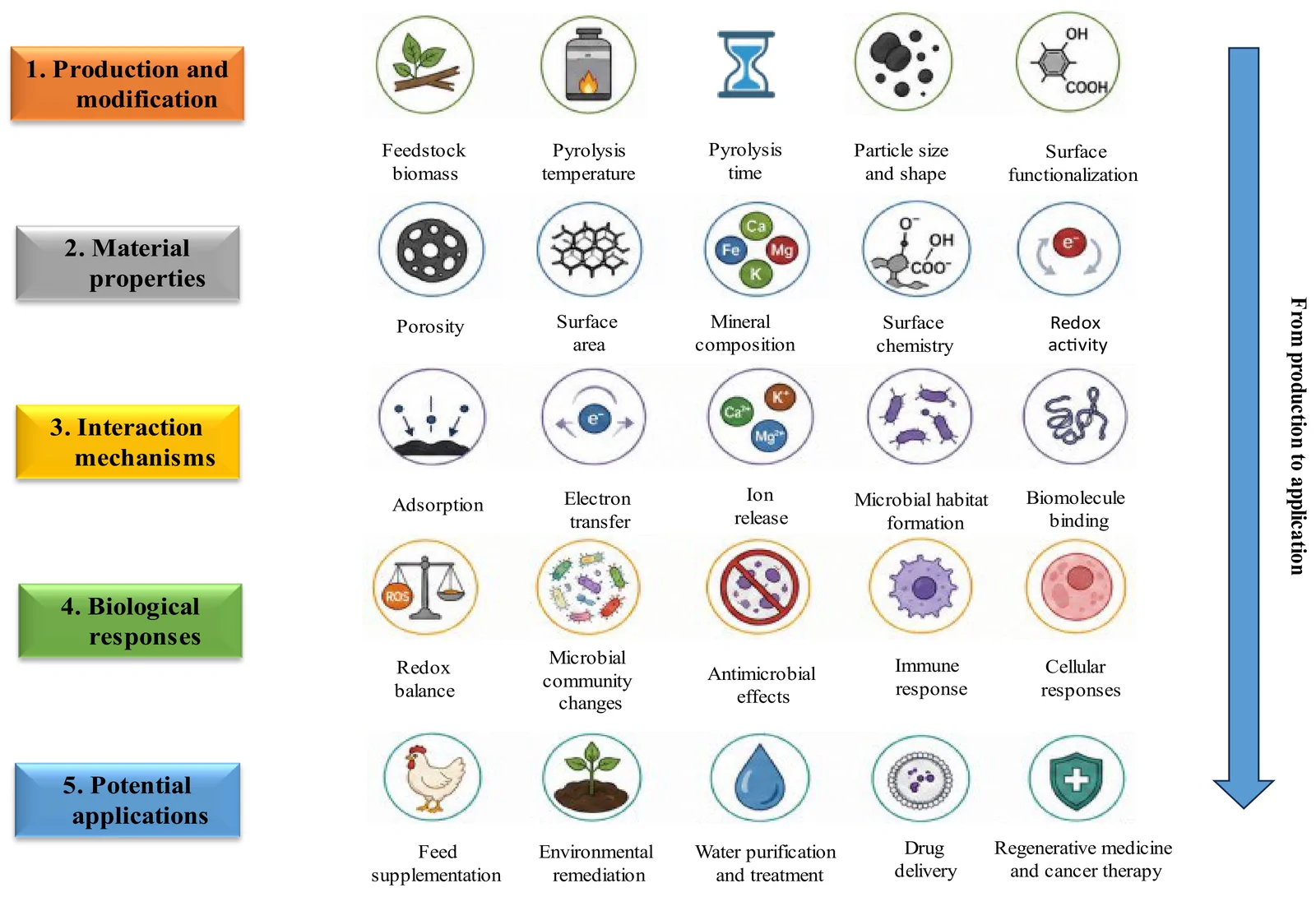
Schematic overview of the production and modification of biomass-derived materials, their physicochemical properties, interaction mechanisms, biological responses, and potential applications.

To ensure safety and optimize performance, standardization, application optimization, and long-term field studies remain essential. Nevertheless, responsibly produced biochar, when integrated into circular-economy and climate-mitigation strategies, may contribute substantially to global greenhouse gas mitigation. For this reason, biochar is considered a promising tool that can help address environmental issues, including agricultural soil amendment, wastewater treatment, cement manufacturing, and the remediation of contaminated soils [54,55]. Figure 1 summarizes the multidisciplinary roles of biochar across environmental, biological, and biomedical systems and related domains. This review builds upon fundamental material properties and extends to advanced applications, including environmental, veterinary, and food-related uses.

### 1.1. Biochar Characteristics, Physicochemical, and Biological Properties

Biochar is commonly characterized by its physicochemical properties, including specific surface area, porosity, surface functional groups, and pH [56]. Biochar has been extensively applied as a soil amendment and environmental material to increase soil fertility, immobilize contaminants (both organic and inorganic), and enhance long-term carbon sequestration [1,57,58]. Biochar can create favorable microenvironments for soil microorganisms by providing suitable habitats and niches, particularly through its porous structure and associated physicochemical characteristics [24,59]. In addition, the introduction of metabolically available labile compounds from biochar into the surrounding soil can significantly influence soil microbial activity and community structure [60].

Feedstock selection has the largest influence on biochar properties [61], ultimately influencing its applications. Biochars derived from plant-based materials such as wood and sugarcane have a greater capacity to improve carbon storage in tropical soils due to their higher aromatic structure, higher carbon concentration, lower H/C ratio, and Fourier transform infrared spectroscopy (FTIR) spectral features compared to nutrient-rich biochars [62]. Animal bone-derived biochar can improve soil quality and reduce Cd and Zn phytoavailability [63], while animal manure-derived biochars are generally rich in phosphorus [64], which can improve nutrient retention [65]. A recent large-scale meta-analysis further confirmed that feedstock type and composition account for the greatest variation in biochar surface area, total and fixed carbon content, pH, cation exchange capacity, ash content, nitrogen content, biochar yield, H/C ratio, and O/C ratio. This variation in properties leads to different applications of biochar; e.g., sludge and manures can be important feedstocks for carbon sequestration purposes, and biochar from crop residues is suitable for improving key soil properties [66].

**Figure 2:**
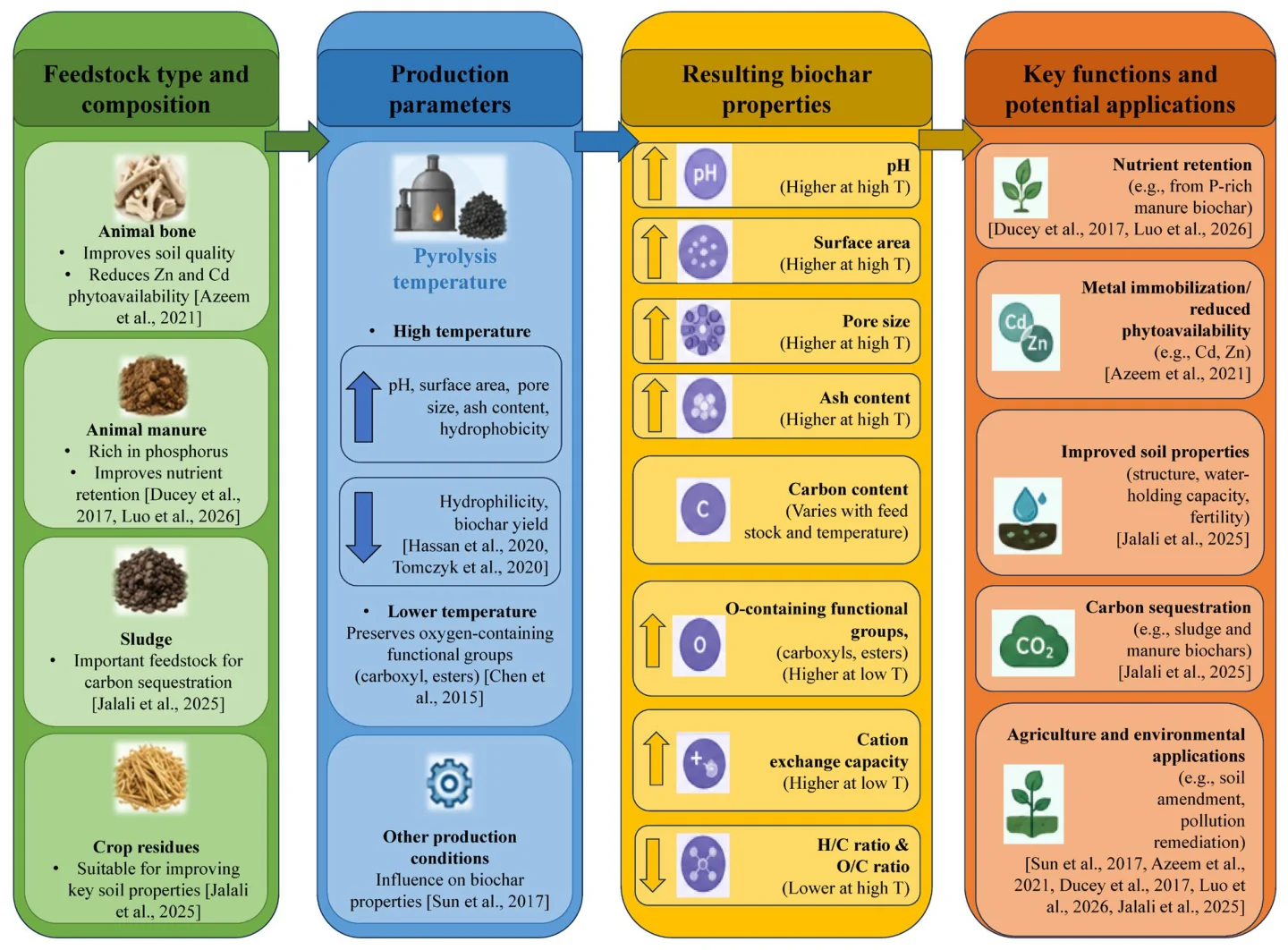
Relationship between biochar feedstock and production parameters resulting in the physicochemical properties and potential environmental applications. The relationships shown are based on experimentally reported findings from the cited literature [13,63,64,65,66,67,68,69].

Production parameters also largely affect biochar properties [13]. High-temperature pyrolysis increases biochar pH, surface area, pore size, ash content, and hydrophobicity, whereas it decreases hydrophilicity and biochar yield [67,68]. In contrast, lower temperatures preserve more oxygen-containing functional groups (carboxyl and esters), which contribute to cation exchange capacity and sorption behavior [69]. The relationships between biochar feedstock, production parameters, the resulting physicochemical properties, and potential environmental applications are summarized in Figure 2.

Biochar possesses a hierarchical pore structure comprising micropores, mesopores, and macropores, as defined by the International Union of Pure and Applied Chemistry (IUPAC) classification [70]. Chemically, biochar is mainly composed of carbon, hydrogen, and oxygen, with variable nitrogen content and an additional mineral (ash) fraction depending on the feedstock [61,71]. Its atomic H/C [72] and O/C ratios are widely used as indicators of biochar stability, compared with production parameters (e.g., pyrolysis temperature and biomass type) or volatile matter determinations [73]. The distribution and type of surface functional groups include aliphatic and aromatic groups [74]. Besides these, surfaces may include hydroxyl, epoxy, carboxyl, acyl, carbonyl, ether, ester, amido, sulfonic, and azyl groups, which strongly affect surface charge, hydrophilicity, cation exchange capacity, and, in general, surface reactivity [75].

Collectively, these physicochemical properties define biochar’s biological activity by governing its interactions with cells, microorganisms [76], biomolecules [77], and dissolved ions [78]. Therefore, the physicochemical properties of biochar determine the application of this biomaterial as an additive to improve soil quality and soil fertility [68]. It is widely known for its sorption capability toward heavy metals such as Cd, Cu, Pb, and Zn [79,80], nutrients such as phosphate [81], and organic contaminants such as pesticides [82] and pharmaceuticals [83]. Biochar sorption takes place through different mechanisms, including physical adsorption, partitioning [31], complexation, physical sorption, precipitation, and electrostatic interactions [84]. Biochars derived from animal-based feedstock are characterized by high calcium-phosphorus content [85] and alkaline pH [86], which improve metal immobilization and nutrient retention, thus enabling diverse applications of biochar in environmental and agricultural systems [85,86]. In soil environments, biochar plays its role in contaminant immobilization and nutrient retention [87], while its broader biological functions are discussed in later sections.

### 1.2. Antioxidative Action of Biochar-Influence on Cell Viability

Reactive oxygen species (ROS) function in a diverse array of signaling pathways when produced at lower, regulated levels in controlled concentrations. However, when oxidants, such as hydrogen peroxide and superoxide anions, are produced in large amounts, they can damage cellular components [88,89]. Biochar is recognized as a functional material capable of influencing redox conditions through its pool of redox-active moieties, including electron-donating, electron-accepting, quinone, and phenolic groups [21]. These properties facilitate the use of biochar as a soil ameliorant by immobilizing environmental contaminants, including heavy metals such as Cd, Cu, and Pb, and reducing their bioavailability [90,91]. These properties also enable biochar to act as an electron donor, electron acceptor, or electron shuttle in processes such as pollutant degradation, metalloid immobilization, and nutrient transformation [92]. Recent mechanistic evidence indicates that biochar redox behavior is governed by its intrinsic electron exchange capacity and the nature, distribution, and accessibility of redox-active surface moieties, which regulate electron-transfer reactions and can influence ROS generation [93,94,95]. Surface redox-active functional groups, particularly quinone and phenolic moieties, can mediate reversible electron-transfer reactions, thereby influencing the redox transformations and oxidative conditions of environmental systems [21,92]. Reductions in metal mobility and bioavailability following biochar addition have been associated with alleviated metal toxicity and improved biological performance in contaminated soils, including increases in microbial biomass and abundance, shifts in microbial community composition, and enhanced microbial activity and functional efficiency [96,97,98]. Under anaerobic conditions, biochar can facilitate direct interspecies electron transfer (DIET) by acting as a conductive carbon conduit that promotes electron exchange between microbial species, thereby enhancing microbial attachment and syntrophic interactions [99]. In addition, biochar amendments during the anaerobic digestion of food wastes may improve process stability by enhancing volatile fatty acid degradation and methane production [100]. Furthermore, biochar has been shown to alleviate antibiotic-associated microbial stress by reducing antibiotic resistance gene (ARG) expression, contaminant bioavailability, and altering ROS genes and SOS-response pathways [101,102].

Evidence from soil-plant studies also supports these mechanistic pathways, showing that biochar application in stressed soils enhances antioxidant enzyme activity and reduces oxidative stress, leading to the improved dry weight of roots, shoots, and seedlings of soybean [103]. Wheat grown under salt stress showed a positive effect of soil supplementation with biochar on plant growth and mitigation of the negative effect of salinity, with a possibility of enhanced crop production [104]. Similarly, in cadmium-stressed maize, the combined application of biochar and gibberellic acid (GA3) enhanced several growth and biochemical parameters, including germination, root and shoot length, chlorophyll content, and protein, phenolic, and malondialdehyde contents in the leaves, roots, and shoots. The amended soils also exhibited increased resistance to heavy metal toxicity, highlighting the potential of biochar and GA3 as a viable strategy for improving maize growth under cadmium stress [105]. Collectively, these findings imply that biochar may reduce oxidative stress in plants through redox-mediated mechanisms. However, further investigation into its interactions with biological systems and its cytotoxic safety is needed to better understand its broader biological outcomes. Although some studies have examined different biochar application rates, the available evidence does not consistently establish a dose–response relationship or a defined toxicity threshold for biochar-mediated redox effects. The concentration range at which these effects may shift from beneficial to potentially adverse therefore remains insufficiently defined and requires further systematic investigation.

### 1.3. Immunomodulatory Action of Biochar

Biochar has been reported to influence plant defense-related mechanisms, including a reduced incidence of disease, suppression of plant pathogens, and enhancement of resistance against foliar fungal pathogen infection [106]. Elad et al. [107] described that soil-applied biochar induces systemic resistance to foliar fungal pathogens on pepper and tomato and to the broad mite pest on pepper. These induced systemic resistance (ISR)-like responses are associated with the increased activity of defense-related enzymes such as phenylalanine ammonia lyase (PAL), guaiacol peroxidase (POX), polyphenol oxidase (PPO), and lipoxygenase (LOX) [108]. These findings correlated with alterations in the root-associated bacterial community of greenhouse peppers, shifting the microbiome composition towards plant-growth-promoting bacteria [109] and leading to disease suppression [107].

Biochar and related charcoal-based products support intestinal health by maintaining intestinal morphology and improving intestinal function under dietary stress conditions. For example, piglets fed charcoal powder, including feed supplementation with 3% wood vinegar compound liquid (CWVC), have been reported to support intestinal health by developing larger cells and cells protruding further into the lumen. CWVC might also activate intestinal functions both at the villus and cellular levels [110]. Mekbungwan et al. [111] demonstrated that CWVC might prevent the harmful effects of pigeon pea meal (PM) dietary toxins on intestinal function, resulting in normal growth performance. An important mechanism underlying these effects is the adsorption of contaminants by biochar, which leads to a decrease in their mobility and bioavailability in soil and water environments, thus lowering the ecological risk [112]. Livestock studies suggest that biochar supplementation may support gut health, improve physiological and production performance (e.g., live weight gain, feed conversion efficiency, feed intake), and reduce enteric methane emissions [113,114]. Biochar appears to affect host health mainly through indirect mechanisms. Recent evidence for direct anti-inflammatory or immunoregulatory effects remains limited, highlighting the need for further mechanistic investigations. However, the available evidence is heterogeneous and is derived largely from plant and livestock models, which limits direct extrapolation to human biomedical applications. Differences in feedstock, dose, formulation, and exposure conditions, together with the limited number of studies directly examining immune-cell responses, make it difficult to establish consistent structure–function relationships or define the long-term immunological safety of biochar.

### 1.4. Proliferation and Cell Cycle Regulation Influenced by Biochar

Cell proliferation is an important biological process controlled by the cell cycle and is responsible for clonogenic potential. A clonogenic assay, or colony formation assay, is an in vitro cell survival assay based on a single cell’s ability to proliferate and form a colony [115,116].

In microbial systems, biochar has been shown to influence intercellular signaling and microbial communication through surface adsorption, pore filling, electrostatic interactions, ion-grid formation, and covalent bonding [117]. Biochar adsorbs quorum-sensing signaling molecules, thereby interfering with bacterial communication and disrupting the coordinated gene expression required for collective microbial behaviors [118,119].

In plants, evidence suggests that biochar influences proliferation-associated signaling. Tan et al. [120] revealed that integrated transcriptomic and metabolomic analyses in *Echinacea purpurea* showed that biochar amendment significantly promoted plant growth and improved nutrient status and was associated with a significant enrichment of plant hormone signal transduction pathways, particularly those of auxin and jasmonic acid (JA), which are key regulators of plant growth, development, and stress [121,122]. Activation of the JA pathway was particularly associated with the simultaneous upregulation of its biosynthetic and metabolic genes, along with a marked accumulation of JA, jasmonoyl-isoleucine, and 12-hydroxy-jasmonoyl-isoleucine [120]. These integrated results show that biochar application can simultaneously promote growth and enhance the secondary metabolism of *E. purpurea* by coordinating phytohormone signaling and phenylpropanoid biosynthesis. These transcriptional changes indicate that biochar may influence the coordination between growth regulation and broader metabolic reprogramming in plants [120].

Engineered biochar-based systems can decrease the proliferation of cancer cells, primarily when used as carriers of bioactive compounds [123]. Fei et al. [123] demonstrated that tailor-made biochar enhanced the antiproliferative activity of butyrate-glyceride (BMDG) formulations against in vitro models of human colorectal cancer cells, i.e., HCT116 and HT29, resulting in reduced cell proliferation and altered cell-cycle progression. Rather than exerting a direct cytotoxic effect, biochar acted as a functional carrier that improved the biological performance of BMDG, likely through its porous structure, adsorption capacity, and controlled release of bioactive metabolites. The enhanced activity depended strongly on the physicochemical properties of the biochar, particularly its meso- and macroporous architecture, highlighting the importance of pore structure and surface accessibility in regulating biological responses [1,123]. Collectively, all of these findings from microbial, plant, and human in vitro systems imply that biochar may affect the proliferative behavior mainly by altering the extracellular, hormonal, and metabolic signaling pathways rather than directly acting on conserved cell-cycle machinery.

Engineered biochar may influence cellular responses, with effects enhanced by material properties such as surface functionalization, particle size, and chemical reactivity rather than bulk composition alone. Further work is therefore needed to develop optimized physicochemical parameters, safe exposure thresholds, and reproducible experimental frameworks to better define how biochar affects proliferative behavior in biological organisms.

### 1.5. Anti-Cancer Properties of Biochar

Nanobiochar (NBC) is produced from biochar by fragmentation into very fine particles via approaches including ball-milling, centrifugation, sonication, and hydrothermal synthesis. It is an advanced nanosized biochar (which can also be modified or engineered to obtain “engineered nanobiochar” or biochar nanocomposites) with enhanced properties and wide applicability [124]. NBC is an excellent candidate for healthcare applications and targeted drug delivery in cancer research because of its versatile properties, like many-fold enhancements in surface area, pore size, total pore volume, and surface functionalities over bulk biochars [124]. NBC greatly improves the water dispersion stability of cancer-targeted drug delivery systems (DDSs) and is a nanomaterial effectively used as a nanocarrier for poorly water-soluble drugs [42]. The importance of surface modification is highlighted by a study showing that orange-peel-derived NBC functionalized with tumor-targeting ligands, including biotin, riboflavin, folic acid, and hyaluronic acid, achieved enhanced cellular uptake of anticancer drugs. By internalizing the poorly water-soluble drug DHF into cancer cells, likely through receptor-mediated endocytosis, the functionalized NBC induces cell death, selective cytotoxicity, and potent in vitro antiproliferative activity against A549 lung cancer cells. Notably, biotin-functionalized NBC resulted in cell death percentages that were more than double in comparison to those achieved by the drug alone. These findings demonstrate the potential of surface-modified NBC for precision oncology applications [42].

Beyond its drug-delivery function, biochar may exert biological effects through redox-mediated mechanisms. Pyrolysis-derived biochar can retain environmentally persistent free radicals (EPFRs) and redox-active surface moieties that promote ROS generation, including superoxide and hydroxyl radicals [125,126,127]. This ROS-generating activity may induce localized oxidative stress and contribute to apoptosis and tumor-cell growth inhibition. Recent studies have reported cytotoxic and antitumor effects of biochar-based materials in colorectal cancer and Caco-2 cells [123,128]. Such ROS-mediated processes are consistent with the principles underlying chemodynamic cancer therapy [129].

Functional versatility can be achieved through surface functionalization and drug-loading strategies, which enable NBC to act as a targeted nanocarrier for anticancer therapeutics [42]. In particular, the combination of silver nanoparticles (AgNPs) and biochar showed antiproliferative properties; they significantly reduced the vascular endothelial growth factor (VEGF) and proinflammatory cytokine expression levels [130]. Furthermore, it also demonstrated reduced expression of anti-apoptotic genes and pro-inflammatory cytokines compared to individual treatments [131]. The enhanced surface functionalities and favorable colloidal stability of NBC can reduce particle agglomeration and improve dispersion stability in aqueous systems [132]. This enhances therapeutic performance without substantial increases in material complexity while maintaining a relatively simple platform design.

Despite these advances, the clinical translation of NBC remains challenging because feedstock composition and pyrolysis conditions can substantially affect specific surface area, elemental composition, morphology, functional groups, and the crystalline and amorphous nature of the carbon, leading to variability in biological performance [132,133]. A recent study by Singh et al. [134] further identified nanobiochar (nanoscale biochar) and biochar nanocomposites, sustainable carbon-based nanomaterials with numerous applications, including the use of biochar nanocomposites for targeted drug delivery and biosensors, which are promising applications for One Health (which integrates human, animal, and environmental health). Nevertheless, clinical translation remains limited by insufficient data on reproducibility and long-term safety, highlighting the need for standardized manufacturing and toxicological evaluation. Despite the growing potential in various applications of nanobiochars, there are still concerns regarding their health effects. For instance, a study by Dong et al. [135] demonstrated that nano-tobacco stem-pyrolyzed biochars (nTSBs) can cause evident cytotoxic and genotoxic effects in epithelial cells (a normal human in vitro model) by inducing ROS formation. nTSB exposure also led to the activation of inflammatory factors. Furthermore, the integrity of the epithelial barrier was also impaired. This study also showed that nTSB exposure increases the risk of chronic obstructive pulmonary disease. However, all of these adverse responses were induced by high concentrations of nTSBs. Accordingly, the pulmonary toxic potential of nTSBs occurring at high concentrations is likely not the same as that occurring at more realistic outdoor exposure concentrations. As the toxicity of nanobiochar to human and ecosystem health has not received sufficient research attention, it highlights the significance of standardized manufacturing and safety protocols [132]. Detailed toxicological evaluation, reproducible synthesis routes, and strict control of redox-active components are therefore important for advancing NBC toward clinical use [132,135]. Importantly, the contribution of biochar itself is not always distinguishable in multifunctional systems containing drugs, nanoparticles, or other active components. Appropriate biochar-only and component-specific controls are therefore needed to clarify its independent biological contribution. More broadly, the heterogeneity of the available anticancer studies limits the interpretation and comparison of their findings. The evidence remains predominantly preclinical and involves heterogeneous nanobiochar formulations and multifunctional composites, with differences in material properties, dose, and experimental models. Long-term safety and biodistribution also remain insufficiently evaluated. Therefore, standardized materials, appropriate controls, and systematic long-term in vivo studies are needed before clinical translation.

Collectively, the convergence of targeted drug delivery, photothermal responsiveness, and redox-based therapeutic mechanisms, together with the sustainability and cost advantages of biomass-derived nanomaterials, highlights the potential of nanobiochar as a promising platform for next-generation cancer therapeutics, although further research is needed to establish its safety, reproducibility, and clinical applicability.

### 1.6. Pro-Regenerative Properties and Applications of Biochar

Regenerative biomedicine (i.e., regenerative medicine) is an interdisciplinary field that is focused on restoring, replacing, or regenerating human cells, tissues, or organs to re-establish normal function through biological and engineering approaches [136]. In this context, biochar has gained attention as a biomaterial whose structural and chemical properties support a range of regenerative processes [134]. Novel and tissue-compatible three-dimensional bioscaffolds can be derived from biochar-based nanocomposites, provide structural support for cell growth, and can be functionalized to facilitate the slow release of molecules, such as growth factors, thereby accelerating tissue proliferation and wound healing. Similar applications of biochar nanocomposites can be found in wound dressings or as coatings in medical devices to prevent infections. Moreover, biochar-incorporated hydrogels, enriched with nanoparticles such as Ag-NPs, can also be used in dressings for wound healing [134,137,138].

The osteogenic activity (osteoconductive and osteoinductive activity) of biomaterials can be enhanced by the release of bioactive mineral ions such as calcium, phosphate, and magnesium, which regulate bone regeneration and bone repair processes [139,140]; biochar naturally contains these mineral constituents, which contribute to its bioregenerative properties and functionality [141]. The incorporation of biochar into hydrogels also improves their regenerative abilities by developing multifunctional wound dressings. Chu et al. [137] developed a photothermal N-halamine hydrogel loaded with biochar produced from cow dung, in which cow dung was first converted into biochar by pyrolysis, which was then coupled with a 3D interpenetrating hydrogel network to treat infected wounds. The hydrogel exhibited excellent synergistic antibacterial performance against *Escherichia coli* (*E. coli*) and *Staphylococcus aureus* (*S. aureus*) within 60 min. The *S. aureus*-infected wound model in vivo demonstrated that the hydrogel worked as an ideal wound dressing, capable of resisting bacterial infection, accelerating the healing process, and promoting epithelial regeneration. Borjihan et al. [138] also formulated a cow dung biochar-based hydrogel for the in situ generation and controllable release of active iodine. This hydrogel showed controllable antibacterial properties and excellent biocompatibility. It can also accelerate the healing of methicillin-resistant *Staphylococcus aureus* (MRSA) infected wounds. Because these wound-healing systems combine biochar with hydrogels and other active components, the specific contribution of biochar to the observed therapeutic effects requires confirmation using appropriate comparative controls. These composite systems exhibit properties including tissue adhesiveness, antibacterial activity, self-healing, and enhanced chronic recovery processes. Altogether, they support the shift from early inflammation to proliferation, which is very important for effective wound repair [137,142]. Collectively, these findings highlight the multifunctional role of biochar as a regenerative biomaterial that integrates infection control, modulation of redox balance, structural support, and physicochemical signaling. Future developments may include macrophage-modulating biochars, ion-doped architectures to improve angiogenesis, and stimuli-responsive delivery systems triggered by pH or enzymatic activity, thus positioning biochar as a tunable material for next-generation regenerative therapies. However, the regenerative effects of biochar-based materials should be interpreted with the consideration of biochar dose and overall formulation composition. The available evidence does not consistently establish dose–response relationships or toxicity thresholds, particularly for biochar-containing hydrogels and nanocomposites, highlighting the need for systematic studies using appropriate dose ranges and component-specific controls. Moreover, these findings remain largely preclinical, and the limited number of in vivo studies makes it difficult to assess the reproducibility and long-term regenerative safety of biochar-based materials.

### 1.7. Antibacterial Activity of Biochar

Recent advances in composite and functionalized biochar materials have enabled their use in antibacterial treatments. Biochar can be functionalized with metal oxides, silver nanoparticles, or polymers to enhance its antimicrobial activity, offering alternatives to conventional antibiotics [143]. Metal-impregnated biochars containing Cu, Fe [144], or Ag [145,146,147,148] show antibacterial performance. Hu et al. [144] developed a magnetic biochar-supported Fe^0^/Cu^0^ composite (BC@Fe^0^/Cu^0^) that effectively inactivated *Escherichia coli* and *Staphylococcus aureus* through carbothermal reduction. The biochar matrix facilitated the formation of active Fe^0^ and Cu^0^ species, enabling strong antibacterial activity with complete *E. coli* inactivation and 97.05% *S. aureus* reduction within one hour.

Recent studies in silver-engineered biochar composites have demonstrated their potential as highly effective antibacterial materials against clinically and environmentally relevant pathogens. Zhao et al. [145] developed a porous silver-loaded biochar composite (p-PVA/C–Ag) that exhibited strong antibacterial activity by effectively inhibiting *Escherichia coli* growth. In another work, Eltaweil et al. [146] developed another Ag@biochar nanocomposite that exhibited broad-spectrum antimicrobial activity, completely inhibiting *Escherichia coli* growth and suppressing the growth of *Pseudomonas aeruginosa*, *Klebsiella pneumoniae*, and *Bacillus subtilis*. In a study by Xie et al. [147], silver-carrying biochar-polyvinyl alcohol-alginate gel beads (C/PVA/SA) were designed for suppressing bacteria. It was found that C/PVA/SA gel beads had a strong inhibitory effect on *Escherichia coli*, *Staphylococcus aureus*, and *Pseudomonas aeruginosa*. Zhang et al. [148] synthesized a nanosilver antibacterial composite derived from fish-scale charcoal and evaluated its activity against Escherichia coli, Staphylococcus aureus, and Pseudomonas aeruginosa. The composite achieved an antibacterial rate exceeding 99.9% against the tested bacterial strains, supporting its potential for the development of antimicrobial coatings and infection-control materials.

Magnetic biochar conjugated with a quaternary phosphonium salt (MBQ) significantly enhances biochar’s antibacterial performance. MBQ is an effective bactericide against both Gram-positive (*Staphylococcus aureus*) and Gram-negative bacteria (*Escherichia coli*). This antibacterial activity is mainly attributed to increased cell damage, cell membrane disruption, and oxidative stress induced by the germicidal agent phosphonium salt (QPS). The biocide effect is further enhanced by the penetration of MBQ particles through the cell wall, cell membrane, and into the cytoplasm [149]. Thus, modified magnetic biochar is a promising dual-function material for pollutant removal and water disinfection. Fu et al. [150] further investigated the removal of tetracycline (TC) by iron nanoparticles (Fe NP). Fe NP@BC obtained appealing TC adsorption and degradation capacity.

Recent evidence indicates that engineered biochar can effectively mitigate the dissemination of antibiotic resistance genes (ARGs). Zhao et al. [151] demonstrated that incorporating biochar into earthworm ecological filters effectively removed both chromosome- and plasmid-carried antibiotic resistance genes (ARGs) from rural sewage. Biochar was particularly effective against plasmid-carried ARGs, reducing their abundance and inhibiting plasmid-mediated horizontal gene transfer, thereby limiting ARG dissemination. Zhou et al. [152] further demonstrated that a Fe-Mo bimetallic sulfide-coated biochar hydrogel (FeMoS-HC) activated by peroxymonosulfate efficiently inactivated antibiotic-resistant bacteria (ARB), removed both intracellular and extracellular ARGs, and markedly inhibited horizontal gene transfer. These effects were attributed to oxidative degradation mechanisms that also suppressed the expression of key genes involved in ARG transfer. Ma et al. [153] reported that nanoscale zero-valent iron-modified biochars (nZVI-SBCs) also effectively inactivated both intracellular and extracellular ARGs in aquatic environments. This effect was achieved through bacterial cell membrane disruption, intracellular ROS generation, and the degradation of plasmid DNA, thereby inhibiting the horizontal transfer of ARGs. Wang et al. [154] further reported that biochar reduced the abundance and dissemination of ARGs by regulating mobile genetic elements, including prophages, insertion sequences, and transposons. Functional gene analysis further indicated that biochar influenced genetic material synthesis and amino acid metabolism, contributing to the regulation of ARGs.

In addition to oxidative and metal-ion-mediated killing mechanisms, biochar-associated antimicrobial effects can also be caused by the adsorption and pore-filling processes, where the porous structure and surface chemistry immobilize microorganisms and adsorb nutrients required for microbial growth, thereby reducing microbial mobility and proliferation. Walnut-shell-derived biochars produced under controlled pyrolysis conditions have been shown to remove multidrug-resistant bacteria (up to 97%) in experimental filtration systems; additionally, these biochars adsorb active pharmaceutical ingredients (up to 97% of methylphenidate and 88% of the antibiotic clarithromycin), suggesting potential for wastewater treatment applications [155]. Recent evidence suggests that biochar can act as an effective adsorbent, and when functionalized, can serve as a promising antimicrobial material, although further mechanistic studies and application-specific optimization are still required. However, antibacterial efficacy is strongly dependent on the applied concentration, material composition, and exposure conditions, and the available studies do not consistently establish dose–response relationships or defined toxicity thresholds. In particular, the contribution of biochar itself should be distinguished from that of incorporated metals or other antimicrobial agents, while potential cytotoxic or ecological effects at higher exposure levels require further evaluation. Moreover, much of the current evidence is based on engineered or functionalized biochars evaluated under application-specific experimental conditions, which limits direct extrapolation to biomedical use and highlights the need for validation in relevant in vivo models.

### 1.8. Regulation of the Microbiome

The porous structure and high specific surface area of biochar, together with its capacity to adsorb soluble nutrients and organic matter, may facilitate bacterial and fungal colonization by creating favorable microhabitats. This may be particularly relevant to the establishment and persistence of beneficial bacterial and fungal taxa. Importantly, these effects are not necessarily selective and depend on the properties of both the biochar and the surrounding environment. Biochar serves as a protective shield for microbes against natural predators such as mites and nematodes, facilitates microbial growth, colonization, and biofilm formation, thereby increasing the total microbial biomass and activity [117,156,157]. With the formation of such micro-niches, biochar may facilitate the persistence of low-abundance microbial taxa, contributing to shifts in microbial diversity and community composition in amended soils [158]. Apart from passive habitat development, biochar can also act as an ideal microbial carrier (and can even replace commercially produced, non-renewable microbial carrier substrates), thereby improving the development of beneficial microbial inoculants [117]. The effects of biochar on soil microbial communities are well-documented, but variability remains high. Bacterial and fungal communities respond differently to biochar’s physical properties, including pH, feedstock type, pyrolysis conditions, pore structure, and particle size, which can alter the microbial community composition and taxonomic distribution. For example, microbial biomass, prokaryotic abundance, and diversity largely depend on biochar properties and its application rate [158].

The modulatory effect of biochar on the microbiome is defined by its characteristics, like surface area, porous structure, labile C, high pH, and electrochemical properties, determining soil microbial abundance and communities and their mediated nitrogen and phosphorus cycling processes, such as nitrification, denitrification, and phosphorus solubilization [159,160,161]. Meta-analytical evidence indicates that biochar amendments generally increase microbial biomass, although variation in responses could be attributed to heterogeneity in soil characteristics, biochar properties, or amendment protocols [162]. The microbial community living in and around the biochar particles had significantly lower prokaryotic diversity and higher eukaryotic diversity than the surrounding soil, due to the formation of distinct biochar-associated microhabitats [161,162,163]. These findings are consistent with other studies showing that biochar can produce negative or contrasting effects on microbial diversity under specific conditions. A field study reported that biochar application significantly decreased microbial community diversity, as reflected in the significant decrease in the Shannon diversity of bacterial and fungal communities [164]. Similarly, a long-term study found that biochar increased bacterial α-diversity but decreased fungal α-diversity and significantly altered both bacterial and fungal community structures [165]. Furthermore, a recent meta-analysis showed that the effects of biochar on soil microbial α-diversity depended on application dose, while bacterial β-diversity was significantly shifted at doses above 2%; although the relative abundances of several bacterial groups increased, those of Verrucomicrobia and Basidiomycota declined following biochar application [166].

Another perspective is the capability of biochar to modulate the gut microbiome in animals. Emam et al. [167] demonstrated that 3% biochar supplementation in the diet of broiler chickens positively affected cecal microbiota, increasing beneficial bacteria and reducing harmful bacteria. Furthermore, it significantly improved growth performance, antioxidant indices in both serum and liver, and digestive enzyme activities, particularly amylase activity. Similarly, Zhang et al. [168] reported that 3% dietary biochar supplementation in pigs enhanced immune function by modulating immune-related pathways. Furthermore, biochar supplementation also reduced mortality and improved the average daily weight gain, leading to better overall growth performance in growing pigs. On the other hand, Tichá et al. [169] demonstrated that feeding 1% biochar as a feed supplement to horses does not affect fecal bacterial community diversity or the relative abundances of major bacterial taxa, showing limited microbiome-modulating potential at the practical inclusion level. It provides clear evidence that the microbiome response is species- and dose-dependent. Collectively, these studies provide evidence that biochar functions not only as an adsorbent in the gastrointestinal tract, but also as a regulator of gut microbial ecological diversity, highlighting its potential as a sustainable feed additive to improve animal health and productivity. Despite ongoing progress, critical knowledge gaps remain, including limited long-term field evidence and insufficient understanding of sorption–desorption dynamics that affect microbial metabolism.

### 1.9. Biochar as a Functional Feed Additive

Biochar itself, as well as its applications, has been evolving in recent years, and currently, it has been used as an innovative functional feed additive. According to meta-analytical evidence provided by both in vitro and in vivo experiments, feed supplementation with biochar improves animal performance, growth rate, feed digestibility, feed conversion rates, body weight gain, microbiota production in the rumen, and reduction in enteric methane production [170,171]. Appell, M. et al. [172] extensively investigated the mechanisms of biochar’s ability to adsorb undesirable compounds such as mycotoxins, e.g., aflatoxin B_1_, ochratoxin A, and zearalenone, at the in vitro level. It may decrease their bioavailability and reduce negative impacts on gut health and nutrient utilization. Simultaneously, its porous structure enhances in vitro ruminal microbial activity and microbial biomass, thus promoting a healthier gut microbiome [173]. Consistent with these findings, an in vivo broiler study demonstrated that dietary biochar alleviated aflatoxin B1-induced impairments in growth performance, immune function, blood indices, and liver histopathology, supporting its potential to reduce mycotoxin toxicity in animals [174]. Although animal studies have shown that dietary biochar is safe and may improve gastrointestinal function and animal performance [175], direct in vivo evidence confirming biochar’s sequestration of mycotoxins remains limited.

Teoh et al. [176] investigated the effect of supplementing the diet with hardwood biochar using the in vitro RUSITEC (rumen simulation technique) system. They concluded that hardwood biochar had no influence on ruminal fermentation characteristics and may potentially decrease the concentration of enteric CH_4_ when included at higher dosages by manipulating ruminal microbiota abundances. The study by Lind et al. [177] investigated both in vitro (rumen fluid from Holstein cows) and in vivo (sheep and lamb) models. This study demonstrated that biochar supplementation had no favorable impacts on in vitro and in vivo CH_4_ production, while there was an increase in the total short-chain fatty acid (SCFA) concentration. Ni et al. [178] studied an in vivo model and demonstrated that enriched biochar did not reduce methane emissions from Holstein steers, and fermentation end products were not affected by dietary strategy. The results related to fermentation effects have motivated an investigation into biochar as a tool for decreasing enteric methane emissions. However, experimental results are contradictory, as indicated by the in vitro and in vivo experimental results. Nevertheless, the meta-analytic evidence by Qomariyah et al. [171] generally states that biochar supplementation of ruminant diets modulates rumen fermentation by increasing propionic acid but decreasing methane emissions.

**Figure 3:**
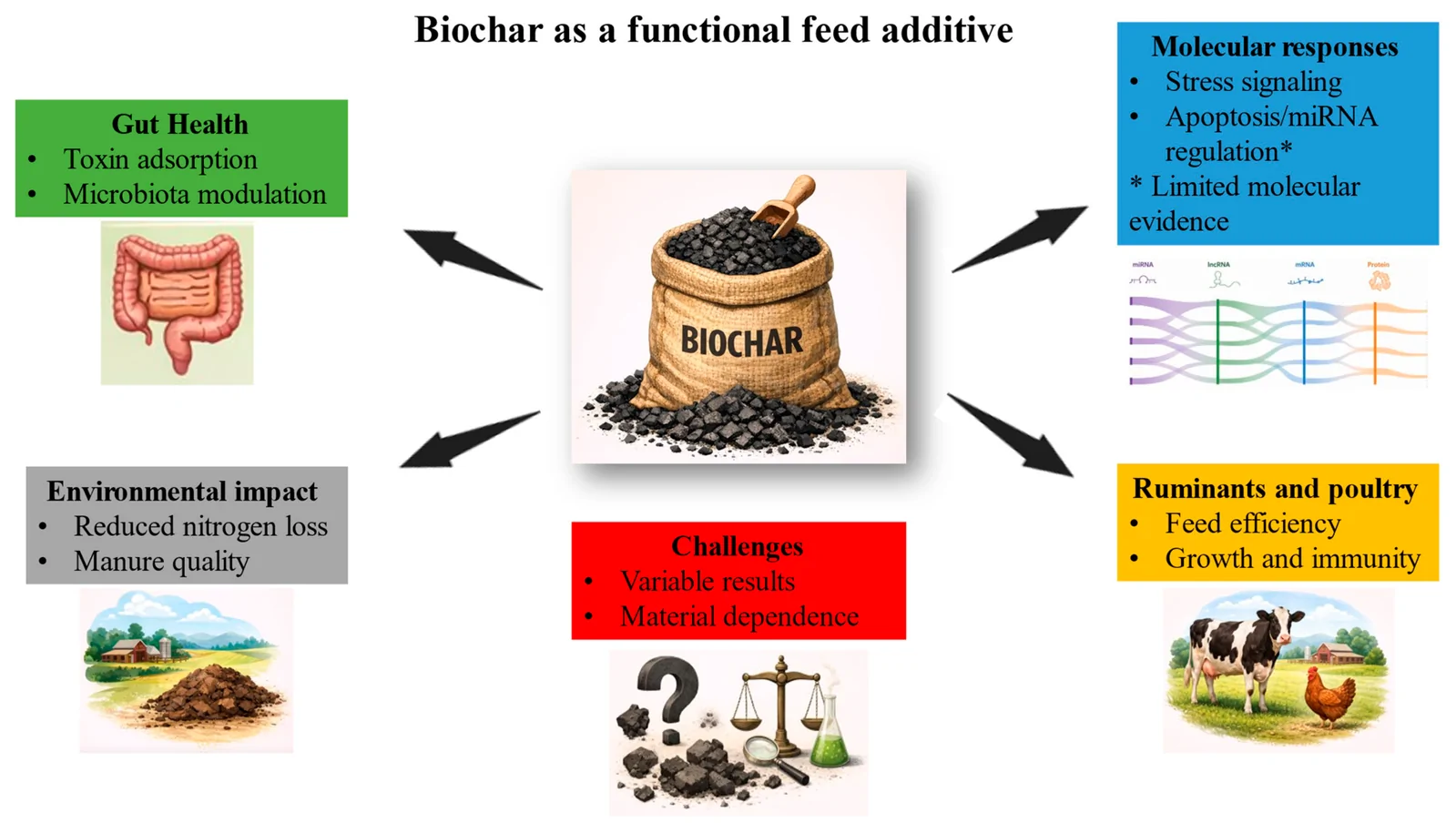
Proposed mechanisms of dietary biochar as a functional feed additive in animal systems.

Molecular studies suggest that biochar can elicit biological effects beyond adsorption and fermentation modulation. Masiello et al. [119] provided evidence that biochars (biochar prepared at 700 °C (surface area of 301 m^2^/g) inhibited cellular communication 10-fold more than an equivalent mass of biochar prepared at 300 °C (surface area of 3 m^2^/g) and had a range of effects on gene expression dependent on intercellular signaling. This affects the bioavailability and activity of quorum-sensing molecules taking part in microbial communication. Quorum sensing regulates virulence expression, biofilm formation, and metabolic coordination [119]. In a transcriptomic study using the model organism *Caenorhabditis elegans*, Chen et al. [179] showed that exposure to biochar led to the differential expression of genes involved in cuticle/barrier integrity, transcriptional regulation, stress signaling, and oxidative stress defense. Further supporting these mechanistic molecular studies, in a recent in vitro study using Caco-2 intestinal epithelial cells, Amin et al. [128] demonstrated that biochar aqueous extracts induced time-dependent regulation of apoptosis-associated pathways accompanied by the modulation of microRNA expression, suggesting that biochar-derived compounds may directly influence intestinal cellular signaling and gene regulatory mechanisms. It presented proof of the concept that biochar can modulate host gene expression at the molecular level. These findings raise the possibility that biochar-mediated cellular signaling could influence host–microbe interactions in gastrointestinal environments. Collectively, these studies provide evidence that dietary biochar may affect not only physicochemical mechanisms, but also cellular regulatory pathways, particularly host gene expression. Figure 3 illustrates the proposed mechanisms through which dietary biochar interacts with gastrointestinal chemistry and host physiology.

Most of the poultry studies revealed more uniform positive effects on animal performance; for example, dietary supplementation of 1% biochar in the diet of broiler chickens improved feed intake, body weight gain, and feed conversion ratio; nutrient digestibility; and enhanced tibia calcium and phosphorus content [180]. Additionally, it improved footpad health, litter quality, and titer against Newcastle disease and infectious bronchitis disease in broilers [180]. Similarly, Emam et al. [167] recently reported that biochar supplementation in broiler chicks significantly improved growth and feed efficiency. Biochar supplementation also influenced blood biochemical parameters by increasing calcium and phosphorus concentrations and promoted a more favorable cecal microbiota through an increase in beneficial bacteria and a reduction in harmful bacterial populations. In contrast, Kalus et al. [181] reported that dietary biochar supplementation increased the feed conversion ratio in broiler chickens. Beyond productivity improvements, Pervaiz et al. [180] associated biochar supplementation with enhanced immune function in poultry, as evidenced by higher antibody titers and increased thymus and bursa weights, although the underlying molecular mechanisms remain insufficiently understood. The potential benefits of dietary biochar therefore extend beyond enteric methane mitigation and animal growth performance. Its ability to adsorb organic nutrients, including fatty acids, urea, and amino acids, may improve nutrient preservation; however, experimental validation of this mechanism under commercial production conditions remains limited [170]. Recent literature focuses both on the promises and limitations of biochar as a feed additive, highlighting material heterogeneity, nonuniform animal responses, and limited mechanistic clarity as main complications.

### 1.10. Biomineralization

Biomineralization is the process by which living organisms influence the precipitation, synthesis, and organization of mineral materials, and it may be considered an ancient process for the accumulation of metal ions in living systems [182]. This process is responsible for creating nonhomogeneous composites of organic and inorganic compounds that depend on the environment in which they form [183]. It has attracted increasing attention in biochar research, as growing evidence indicates that biochar contains a mineral fraction comprising alkaline and alkaline-earth elements, including Ca and Mg, which may occur in forms such as carbonates, phosphates, hydroxides, and oxides [184,185]. Although conventional biomineralization refers to biologically controlled mineral formation, biochar research has extended this concept to encompass biochar-mediated mineral precipitation and the formation of mineral phases associated with its carbonaceous matrix. In this context, we use “biochar-mediated mineralization” to describe mineral precipitation or transformation associated with biochar surfaces or constituents, whereas “biologically induced mineralization” refers specifically to mineral formation directly mediated by living organisms or their metabolic activity. Rees et al. [186] showed that metals can be immobilized within calcium carbonate crystals (formed from calcium oxalate during pyrolysis), as well as in newly formed carbonate precipitates associated with the original wood structure. The mineralization and metal-immobilization behavior of biochar is influenced by its inherent carbonate phases and their distribution within the biochar structure [186].

**Figure 4:**
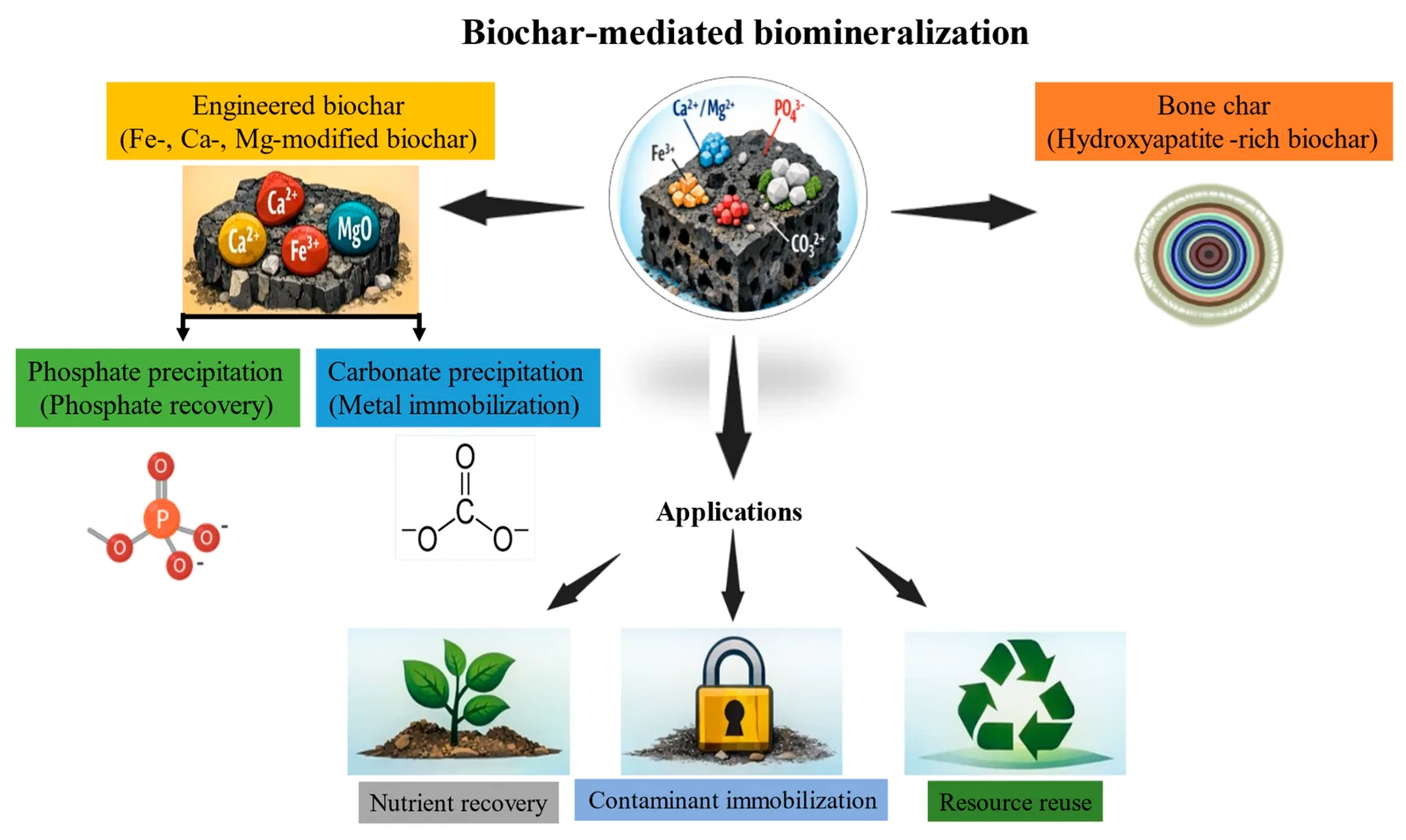
Conceptual illustration of the major biochar-mediated biomineralization pathways and their environmental applications.

Various mineral phases have been incorporated into or associated with biochar through approaches such as co-pyrolysis [187] and post-pyrolysis modification [188]. These include clay minerals [189], sulfides and sulfates [190], phosphates [191], metal oxides [192], and silicate minerals [193]. Mineral-modified biochars have shown considerable promise for soil remediation [194], enhancement of soil fertility [195], wastewater treatment [188], and rainwater management [196]. The major biochar-mediated biomineralization pathways, including phosphate and carbonate precipitation, hydroxyapatite-associated bone char, engineered biochars, and their environmental applications, are summarized in Figure 4.

Heavy metal adsorption takes place through the precipitation of insoluble metal compounds both on the biochar surface (where the biochar acts as a nucleation site for precipitate formation) and in the surrounding solution (through reactions between dissolved metal ions and inorganic species released from the biochar) [197]. Beyond heavy metal immobilization, similar biomineralization processes have been exploited for nutrient recovery, particularly phosphorus, through the formation of stable phosphate minerals on engineered biochars.

Phosphorus (P) is an essential nutrient for life. It sustains the productivity of food systems; yet surplus phosphorus can accumulate in wastewater and can be the cause of water eutrophication if not properly treated [198]. Therefore, phosphorus removal and recovery are receiving increasing attention as important components of sustainable wastewater treatment and nutrient management [198]. Zeng et al. [199] demonstrated that the removal of low phosphate concentrations is primarily driven by the electrostatic attraction (physical adsorption) of phosphate ions to positively charged biochar surfaces, whereas at higher phosphate concentrations, removal is mainly achieved through chemical precipitation, in which phosphate reacts with iron or calcium present in engineered biochar (ZFCO-BC: metal-modified biochar loaded with Fe and Ca) to form insoluble compounds.

Metal-modified biochars, particularly those containing Mg-based minerals such as MgO, have been widely investigated for efficient phosphorus recovery. This can occur through a variety of mechanisms: physical adsorption, precipitation, and inner-sphere surface complexation. These mechanisms lead to the formation of Mg(H_2_PO_4_)_2_ and MgHPO_4_ on the biochar surface, allowing the recovered phosphorus to be reused as a slow-release fertilizer following desorption [200]. Mineral (Fe) modified biochar can also simultaneously recover nitrogen and phosphate from biogas slurry and may subsequently serve as a slow-release fertilizer [201]. Engineered biochars modified with Mg-, Ca-, and Fe-containing phases can alter surface chemistry and promote phosphate removal through adsorption and precipitation processes [199,202,203]. Such approaches allow biomineralization to be tuned toward specific target phases and applications.

Biochar-mediated biomineralization also includes carbonate mineral formation. Metals can be immobilized on biochars by precipitation with carbonate [186]. Biochar is also involved in enhancing microbially induced calcite (CaCO_3_) precipitation, improving cadmium immobilization and soil function through biomineralization processes [204]. These mechanisms demonstrate the multifunctional role of biochar in environmental engineering and soil remediation applications. In addition to engineered mineral phases, naturally occurring minerals also contribute to the biomineralization potential of certain biochars, particularly bone char.

Bone char is a specialized type of biochar that naturally contains hydroxyapatite (calcium phosphate) within its carbonaceous matrix, making it particularly suitable for biomineralization and bone-related applications [205]. The presence of hydroxyapatite provides bioactive properties that support mineral deposition, making bone char particularly attractive for biomineralization and regenerative applications. In regenerative medicine, bone char and hydroxyapatite-containing cryogels have shown both in vitro mineralization potential and in vivo evidence of bone formation. Hixon et al. [206] found that bone-char-incorporated gelatin-chitosan cryogels exhibited appropriate porosity and interconnectivity, adequate swelling and mechanical properties, excellent cell attachment, and increased mineralization, supporting their suitability for bone tissue engineering. Subsequently, Robertson et al. [207] reported woven bone formation associated with bone-char-containing cryogels in a rat calvarial fracture model based on histological and microCT findings, although plain cryogels showed greater overall bone-regeneration capabilities after 8 weeks, highlighting the influence of scaffold composition and organization. Thus, in vitro mineralization should be distinguished from direct evidence of bone regeneration, with the latter requiring a demonstration of new bone formation in an appropriate in vivo model. Collectively, these findings demonstrate that the mineral composition of biochar can be deliberately engineered to promote biomineralization and precipitation processes, thereby expanding its applications in environmental remediation, nutrient recovery, and regenerative technologies.

## 2. Research Gaps and Future Perspectives

Despite the growing interest in biochar and research efforts to explain its properties and applications as reviewed in our manuscript, important knowledge gaps and new research directions must be addressed to improve translational and scalable applications of biochar. Among these gaps, improved standardization and reporting represent a fundamental priority because they underpin reproducible mechanistic studies and meaningful comparison across biological and environmental systems. Mechanistic validation and systematic evaluation of long-term safety and translational applicability should then be pursued using these standardized approaches. The critical limitations include insufficient standardization of biochar materials, diversity in feedstock selection, pyrolysis parameters (time and temperature), and post-processing treatment steps. This leads to a wide range of variability in physicochemical characteristics, redox properties, biological response behavior, and ultimately, the possible applications. This diversity further leads to complications, including difficult cross-study comparisons, limited reproducibility, and blurred structure–function relationships, particularly in biomedical scenarios where precise control over surface chemistry, particle size distribution, and environmentally persistent free radical (EPFR) content is very important. Further research should be focused on harmonized classification frameworks and reference biochars, together with reporting standards that specify key material characteristics, including feedstock and pyrolysis conditions, particle size, surface area and porosity, surface functional groups, pH, elemental and mineral composition, and relevant redox properties of the biochar. Experimental conditions, including biochar concentration, exposure duration, and biological model, should also be reported consistently to facilitate reproducibility and meaningful comparison between studies. Future comparative studies should combine standardized material characterization with controlled experimental designs in which key biochar properties are systematically varied and biological responses are evaluated under comparable exposure conditions. Such approaches would help distinguish effects associated with specific material characteristics from those arising from differences in experimental systems and provide a basis for developing reproducible structure–property–function relationships. Where possible, findings should be validated across independent biochars and complementary biological models to assess the robustness and consistency of the proposed mechanisms. In biomedical applications, these limitations are further compounded by the frequent use of multifunctional composites, in which the independent contribution of the biochar matrix may be difficult to distinguish from that of incorporated drugs, nanoparticles, metals, polymers, or other active components.

While adsorption, redox modulation, and microbial interactions are broadly invoked, a quantitative understanding of these processes remains limited, with effects varying according to feedstock type, production conditions, and biological context [1]. The dynamic balance between reactive oxygen species (ROS) generation and scavenging remains poorly understood and largely depends on the biochar properties and exposure context. Unraveling the conditions under which biochar-mediated redox activity transitions from beneficial to potentially deleterious outcomes remains an important research challenge, particularly for emerging systems with prospective biomedical applications. To address these knowledge gaps, advanced spectroscopic, electrochemical, and in situ imaging techniques should be integrated with biological assays to elucidate electron-transfer pathways, EPFR stability, and the spatiotemporal dynamics of ROS generation in complex biological microenvironments.

Another significant knowledge gap lies in the long-term biological fate and safety of biochar, mainly nanoscale and composite formulations. Although short-term cellular responses to biochar have been investigated, comprehensive data on the effects of chronic or recurrent exposure, tissue accumulation, and long-term biological responses remain limited, particularly for fine and nanoscale biochar particles [208]. This is specifically related to inhalation risks, inflammatory phenomena, immune system interactions, and oxidative stress [208]. Further studies should include comprehensive toxicological assessments and evaluate the effects of realistic long-term exposure to support regulatory approval and the safe use of biochar-based materials. Furthermore, recent analyses emphasize that standardized nanobiochar synthesis protocols, reproducible physicochemical characterization, and harmonized biocompatibility testing are prerequisites for reliable comparison among studies and for the successful clinical translation of biochar-based biomaterials [134].

From an application point of view, incorporating multifunctionality without increasing complexity remains a striking challenge. Biochar’s capability to simultaneously modify redox balance, deliver drugs, inhibit pathogens, and support tissue regeneration is attractive; however, coupling these functions often introduces trade-offs in stability, predictability, and manufacturability.

Design strategies such as surface functionalization, heteroatom/element doping, and composite or coating-based architectures have been widely explored to tailor the physicochemical properties of biochar for environmental and energy applications [209,210,211]. Extending these approaches to the rational design of biochar-based biomaterials represents a promising direction for future biomedical applications; however, advanced functions such as immunomodulation, microbiome modulation, and stimulus-responsive systems remain largely exploratory and require systematic validation.

Finally, future perspectives should focus on translation and sustainability in parallel, leveraging biochar’s low-cost, renewable origins while meeting the stringent performance and safety requirements of biomedical technologies. Future investigations should also prioritize clinically relevant in vitro model studies, pharmacokinetic and biodistribution studies, and scalable manufacturing strategies to facilitate regulatory acceptance and commercialization of biochar-based biomedical technologies [134]. Altogether, addressing these knowledge gaps will enable the shift of biochar from a multifunctional experimental material to a robust, standardized, and application-ready platform for agriculture, environmental management, health, and regenerative medicine.

## 3. Critical Appraisal of the Current Evidence and Its Limitations

The multifunctionality of biochar reflects the interplay between its physicochemical characteristics and the conditions under which it interacts with biological and environmental systems. Although several mechanisms have been proposed, their relative importance remains context-dependent. Among these, biochar can participate in electron donation, electron acceptance, or electron shuttling through redox-active functionalities such as phenolic and quinone-like groups, whose abundance and accessibility are influenced by feedstock and pyrolysis conditions [21,92,93]. These redox properties are also associated with the presence of environmentally persistent free radicals (EPFRs), whose formation is influenced by feedstock and pyrolysis temperature [125,126,127] and which may contribute to ROS generation [125,126]. However, a consistent quantitative relationship between EPFR concentration, redox potential, electron-exchange capacity, and specific biological outcomes has not been established [83,84,85]. Beyond processes associated with the solid biochar matrix, biological effects may also result from compounds released from the material. Evidence from biochar water-soluble fractions, leachates, and dissolved organic matter demonstrates that these released constituents can independently influence biological or environmental processes [50,212,213].

Overall, the current evidence indicates that biochar-mediated mechanisms are strongly influenced by biochar characteristics, dose, production conditions, exposure environment, and the receiving biological system. Consequently, donor or acceptor behavior cannot be predicted solely from production conditions, and universal thresholds or consistent dose–response relationships for redox-related biological effects remain difficult to establish. Moreover, because dissolved or leachable constituents can contribute to observed responses, the relative contributions of the intact biochar matrix and released fractions remain insufficiently resolved across different biochars and biological models. Future studies should therefore use appropriate comparative controls, including biochar-free and dissolved-fraction or leachate treatments where relevant, to distinguish these contributions and establish more robust mechanism-response relationships.

## 4. Conclusions

Biochar has evolved from a conventional carbon-rich soil amendment into a multifunctional material with applications spanning environmental remediation, agriculture, animal production, and emerging biomedical technologies. As demonstrated throughout this review, these diverse applications are governed by the physicochemical properties of biochar, which are determined by feedstock selection, thermochemical conversion conditions, and post-production modifications. Consequently, variations in surface chemistry, hierarchical porosity, mineral composition, and redox-active functional groups directly influence biochar’s environmental reactivity and biological performance. This review highlights that the multifunctionality of biochar arises from fundamental structure-property-function relationships. Surface-mediated adsorption, electron transfer, redox modulation, microbial habitat formation, biomolecule interactions, and biomineralization collectively underpin its roles in oxidative stress regulation, microbiome modulation, antimicrobial activity, tissue regeneration, and targeted therapeutic applications. Furthermore, recent advances in engineered biochars, nanobiochar, and biochar-based composites demonstrate that a rational modification of biochar properties can substantially expand its functional capabilities while maintaining the sustainability advantages of biomass-derived carbon materials. Although significant progress has been achieved, broader implementation of biochar requires improved standardization of production and characterization methods together with a deeper mechanistic understanding of its interactions within environmental and biological systems. Continued integration of environmental chemistry, materials science, microbiology, and biomedicine will enable the rational design of next-generation biochar-based technologies, supporting sustainable solutions for environmental protection, resource recovery, agriculture, and human health.

## Data Availability

The original contributions presented in this study are included in the article/Supplementary Materials. Further inquiries can be directed to the corresponding authors.

## Supplementary Materials

The following supporting information can be downloaded at: https://www.mdpi.com/article/10.3390/molecules31203648/s1, File S1: Review paper methodology; File S2: Characteristics of the biochars and experimental conditions reported in the studies included in this review.
