# Supplementary material for: Biochar Beyond Soil Amendment: Emerging Biological, Environmental, and Biomedical Properties and Applications
Source: Molecules. 2026 Oct 13;31(20):3648. doi: 10.3390/molecules31203648 (PMC13707389; doi:10.3390/molecules31203648)
Supplement: Supplementary file 1 [file molecules-31-03648-s001.zip › File S1. Review Methodology.pdf]

Methodology

This work was conducted using a well-structured, narrative-based literature review developed to thoroughly synthesize current knowledge on the physicochemical properties, biological applications, and evolving biomedical uses of biochar. Based on the multidisciplinary scope of the topic, spanning environmental science, agriculture, microbiology, materials science, and biomedicine, a consistent yet flexible review methodology was chosen to include both foundational studies and recent advances. The overall workflow of the literature search, selection criteria, and thematic synthesis applied in this review is summarized in **Figure S1**.

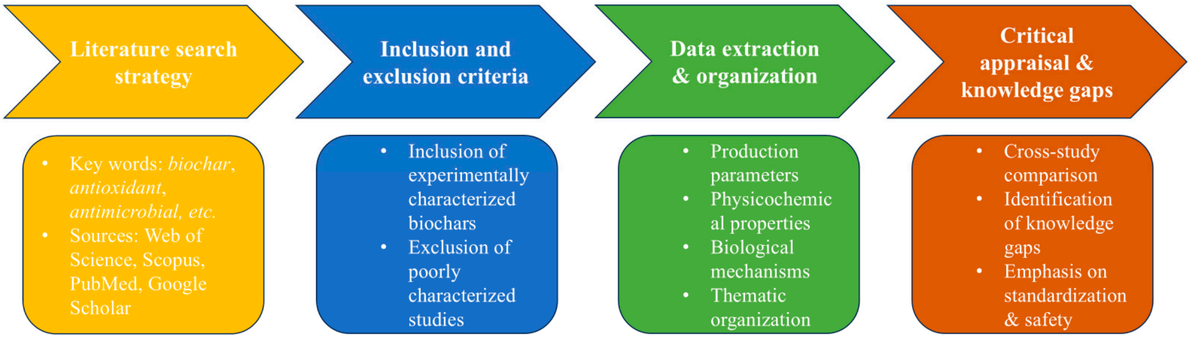

Figure S1. Graphical representation of the review methodology.

Literature search strategy

A literature search was conducted in major scientific databases, including Web of Science, Scopus, PubMed, and Google Scholar. Searches were conducted using combinations of keywords and Boolean operators, including *biochar*, *nanobiochar*, *biomedical applications*, *antioxidant*, *antimicrobial*, *immunomodulation*, *microbiome*, *biomineralization*, *regenerative medicine*, *wound healing*, *bone regeneration*, and *anticancer*. Particular emphasis was placed on recently published studies (2025–2026) to ensure that emerging advances in biomedical applications, mechanistic insights, and translational perspectives of biochar were incorporated into this review. To ensure incorporation of the most up-to-date findings, emphasis was placed on peer-reviewed articles published within the last 15 years, while seminal and historically relevant studies from previous years (older than 15 years) were also included (if needed) to provide conceptual and mechanistic understanding.

Inclusion and exclusion criteria

Inclusion of the studies was carried out based on the following conditions: if they (i) mentioned experimentally characterized biochar or biochar-derived products, (ii) analyzed physicochemical characteristics related to biological interactions, or (iii) evaluated biological, agricultural, environmental, or biomedical applications linked with biochar application. Both *in vitro* and *in vivo* studies were included, along with field-scale assessments where relevant. Reviews, meta-analyses, and authoritative book chapters were considered to contextualize results and find agreement or disagreement within the field. Studies were excluded if biochar characterization was incomplete, if results could not be clearly ascribed to biochar-related mechanisms, or if reports were missing peer review or scientific reliability.

Data extraction and organization

Important data were collected from selected studies, including feedstock type, production method, pyrolysis parameters, post-processing or modification approaches, physicochemical characteristics (e.g., surface area, porosity, elemental composition, surface functional groups), and reported biological effects. Particular emphasis was placed on mechanistic insights linking material characteristics to resulting biological effects, such as redox activity, adsorption behavior, microbial modulation, or cellular responses. The obtained data were organized thematically rather than chronologically to allow cross-comparison across disciplines and application areas.

Thematic synthesis and analytical framework

This review was built around ten thematic areas that consider both the intrinsic characteristics of biochar and its functional biological applications: physicochemical properties; antioxidative action; immunomodulation; antibacterial efficacy; effects on cell proliferation and clonogenic ability; microbiome regulation; biomineralization; anticancer activity; and pro-regenerative uses. This structure allowed integration of environmental and agricultural findings with evolving biomedical research, focusing on shared mechanisms such as redox modulation, surface-mediated interactions, and structure-function relationships. Nanobiochar and engineered biochar composites were considered distinct but relevant subclasses due to their unique size-dependent properties and biomedical relevance.

Critical appraisal and identification of knowledge gaps

In addition to the quantitative analysis, a qualitative critical appraisal approach was also implemented to evaluate the consistency, reproducibility, mechanistic support, and translational relevance of reported results. Inconsistencies among studies were considered in relation to variability in biochar production conditions, material characteristics, experimental models, and reported outcomes. Particular attention was given to whether proposed mechanisms were supported by direct experimental evidence or inferred from associations between biochar properties and biological responses. This approach enabled the identification of key knowledge gaps, including limited standardization of biochar characterization, insufficient long-term toxicity data, and incomplete understanding of the

mechanisms underlying reported biological and environmental effects. Collectively, this methodology provided an integrative framework for assessing the evidence base while considering the limitations affecting interpretation and translation of biochar multifunctionality.
