# Supplementary material for: Biochar Beyond Soil Amendment: Emerging Biological, Environmental, and Biomedical Properties and Applications
Source: Molecules. 2026 Oct 13;31(20):3648. doi: 10.3390/molecules31203648 (PMC13707389; doi:10.3390/molecules31203648)
Supplement: Supplementary file 1 [file molecules-31-03648-s001.zip › File S2. Characteristics of biochars and experimental conditions.pdf]

Table S1. Characteristics of biochars and experimental conditions reported in the studies included in this review.

	Feedstock	Pyrolysis temperature (°C)	Modification	Dose/application rate	Reference
1.	Swine manure	300 and 700	NR	30 g/kg soil (3%, w/w)	Dai et al. [19]
2.	Rice straw	400, 500, 600	NR	0.01 g biochar per experiment	Wang et al. [20]
3.	Grass, wood	200,300,400, 500,600,700		1 or 4 g/L	Klüpfel et al. [21]
4.	Pine wood chips	620	Particle-size fractionation: <0.5, 1–2, and <4 mm	1% and 6% (w/w)	Prodana et al. [22]
5.	Cd-enriched rice straw	300, 400, 500, 600	Separation into macro-, colloidal-, and nano-sized fractions	NR	Gu et al. [25]
6.	Maize straw and peanut hulls	NR	NR	7,800 kg/ha	Ma et al. [26]
7.	Maize and wheat straw	450	NR	20 and 40 t dw/ha	Jílková et al. [27]
8.	Corn straw	450	NR	10, 20, 30 t/ha	Yang et al. [28]
9.	Cottonseed husk (30%) + rice husk (70%)	400	NR	30, 60, 90 Mg/ha	Ding et al. [29]
10.	Wheat straw	350-550	NR	10, 20, 40 t/ha	Bian et al. [33]
11.	Lychee branches (pruning waste)	500	NR	2.5%, 5%, 10%	Jun et al. [34]
12.	Orange peels	240	Yes-NaOH treatment + HNO_3 neutralization/dialysis; subsequent functionalization	NR	Iannazzo et al. [42]

13.	Lolium perenne (perennial ryegrass) shoots	400	NR	108 mg/45 g soil or loess	Kuzyakov et al. [45]
14.	Corncob	230, 369, 416, 562, 600, 796	NR	0.12% or 1.2% dry weight (4 or 40 t/ha), followed by addition of 2.3% dw (78 t ha^{-1})	Budai et al. [46]
15.	*Miscanthus giganteus* straw	230, 235, 369, 385, 416, 503, 600, 682	NR	0.12% or 1.2% dry weight (4 or 40 t/ha), followed by addition of 2.3% dw (78 t ha^{-1})	Budai et al. [46]
16.	Wheat straw, rice husk	550	NR	0, 5, 10, 15, 20% (w/w)	Elliston & Oliver et al. [48]
17.	*Fraxinus excelsior*, *Quercus robur* and *Fagus sylvatica* trunks and large branches	450	NR	2% (w/w)	Xing et al. [49]
18.	Pine (woody feedstock)	300	NR	5, 10, 20% (v/v); coarse biochar also at 10% (v/v)	Ravi et al. [51]
19.	*Schima superba* litter, *Cunninghamia lanceolata* litter	350, 550, 750	NR	20 g/kg soil	Lu et al. [52]
20.	Maize (*Zea mays*) stover, Switchgrass (*Panicum virgatum*),	850	Air-cooled; sieved <2 mm or 2-4 mm; water-doused mixed-size material	1, 10% (w/w)	Clay et al. [53]

	Ponderosa pine (*Pinus ponderosa*)				
21.	Cow bones	500, 800	NR	2.5, 5, 10% (w/w)	Azeem et al. [64]
22.	Pig carcass, *Platanus orientalis* branches	650	NR	0.5, 1, 2, 4% (w/w)	Chen et al. [87]
23.	Wheat straw	~450	NR	5% and 15% (w/w or w/v)	Cui et al. [91]
24.	Maple residues (*Acer pseudoplatanus*)	560	NR	10%, 20% of total pot mass	Farhangi-Abriz & Torabian et al. [104]
25.	Orange and potato waste	325 ± 5	Washing with distilled water (deashing)	0.75% (w/w)	Anwar et al. [106]
26.	Wood/wood charcoal	NR	Wood vinegar compound liquid (CWVC; charcoal:wood vinegar = 4:1)	1, 3, 5% of diet	Mekbungwan et al. [111]
27.	Broadleaf tree wood	300-450	Wood vinegar adsorbed onto amorphous charcoal carbon powder (1:4)	10, 30 g/kg diet	Mekbungwan et al. [112]
28.	Maize straw	450-500	NR	1.3% (w/w)	Tan et al. [121]
29.	Alder wood chips	550	BMDG (butyrate mono-/diacyl-glycerides) absorbed onto biochar	0.25 mg/mL biochar; BMDG-Biochar 0.75 mg/mL	Fei et al. [124]
30.	Beech wood chips, Oak wood chips	600	NR	3 mg/mL biochar-aqueous extract	Amin et al. [130]
31.	Date seeds	550	AgNPs combined with biochar	Biochar: IC50; AgNPs: 3 µg/mL	Abu Hajleh et al. [134]

32.	Willow, sewage sludge, rice husk, oilseed rape	450-750	Ball milling to produce nanobiochar	NR	Raczkiewicz et al. [136]
33.	Tobacco stems	500	Ball milling to produce nano-biochar	NR	Dong et al. [138]
34.	Corn straw	900	Ag loading during carbonization; subsequent incorporation into porous PVA gel	0.2, 0.3, 0.4, 0.5, 0.6, 0.8, 1.0 g/L p-PVA/C–Ag	Zhao et al. [148]
35.	Fish scales	300	AgNP loading; incorporation into PVA/SA gel beads	0.5, 1.0, 1.5, 2.0, 2.5, 3 g/L	Xie et al. [150]
36.	Fish scales	NR	Nanosilver (AgNP) loading onto fish-scale biochar	0.1 g F/C-Ag per well	Zhang & He et al. [151]
37.	Corn straw	500	Magnetic Fe_3O_4 loading followed by quaternary phosphonium salt (QPS) loading	20, 50, 100, 200 mg/L	Fu et al. [152]
38.	Solid digestate	NR	Fe nanoparticle (Fe NP) impregnation	1.0 g/L	Fu et al. [153]
39.	Mango tree branches/ wood	550	NR	1.5% and 3% of diet (w/w)	Emam et al. [170]
40.	Apple tree wood	650	NR	3% of diet (w/w)	Zhang et al. [171]
41.	Wood	300, 700	NR	1% (w/v)	Masiello et al. [182]
42.	Excess activated sludge	600	Mg modification; Mg-Fe double-oxide loading	NR	Zhang et al. [206]
